# Supplementary material for: Cardiovascular and renal effects of apelin in chronic kidney disease: a randomised, double-blind, placebo-controlled, crossover study
Source: Nat Commun. 2024 Oct 14;15:8387. doi: 10.1038/s41467-024-52447-7 (PMC11473822; doi:10.1038/s41467-024-52447-7)
Supplement: Supplementary file 1 — Supplementary Information [file 41467_2024_52447_MOESM1_ESM.pdf]

Supplementary Information

Cardiovascular and renal effects of apelin in chronic kidney disease: a randomized, double-blind, placebo-controlled, crossover study

Fiona A. Chapman[1,2]
Vanessa Melville[1]
Emily Godden[1]
Beth Morrison[1]
Lorraine Bruce[1]
Janet J. Maguire[3]
Anthony P. Davenport[3]
David E. Newby[1]
Neeraj Dhaun[1,2]

[1]University/BHF Centre for Cardiovascular Science, The Queen's Medical Research Institute, University of Edinburgh, Edinburgh, UK.
[2]Department of Renal Medicine, Royal Infirmary of Edinburgh, Edinburgh, UK
[3]Division of Experimental Medicine and Immunotherapeutics, Addenbrooke's Centre for Clinical Investigation, University of Cambridge, UK.

Correspondence to: Dr Neeraj Dhaun *(Bean)*
Edinburgh Kidney
University/BHF Centre for Cardiovascular Science
Queen's Medical Research Institute
47 Little France Crescent
Edinburgh, EH16 4TJ
Telephone: +44 131 242 3100
E-mail: bean.dhaun@ed.ac.uk

Short title: Apelin in kidney disease

Contents *Page*

1. *Supplementary figures* 2
2. *Statistical model outputs* 10

SUPPLEMENTARY FIGURES

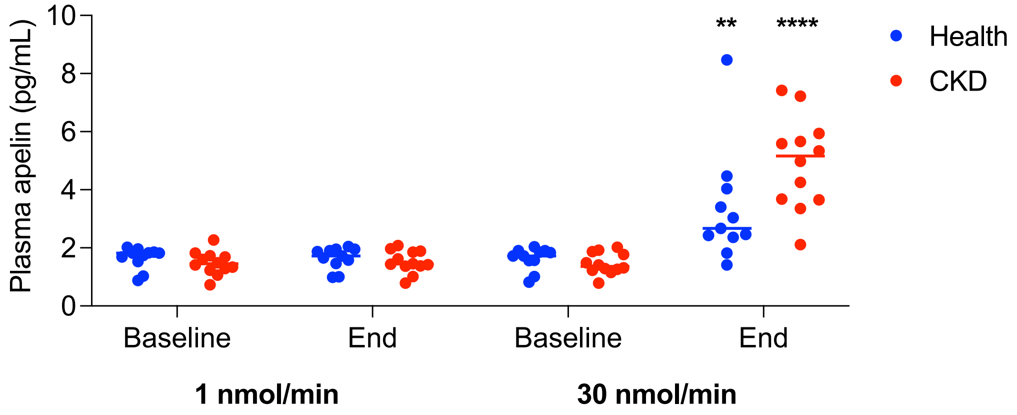

Supplementary figure 1. *Change in plasma apelin concentration following infusion of [Pyr[1]]apelin-13.* Circulating apelin concentration did not change with infusion of 1 nmol/min [Pyr[1]]apelin-13 but rose significantly with infusion of 30 nmol/min [Pyr[1]]apelin-13 in healthy subjects (blue, n=12) where **p=0.009 and in chronic kidney disease (CKD, red, n=12) where ****p<0.0001 for comparison to respective baselines. Line at median. Analysis by two-way paired t-test.

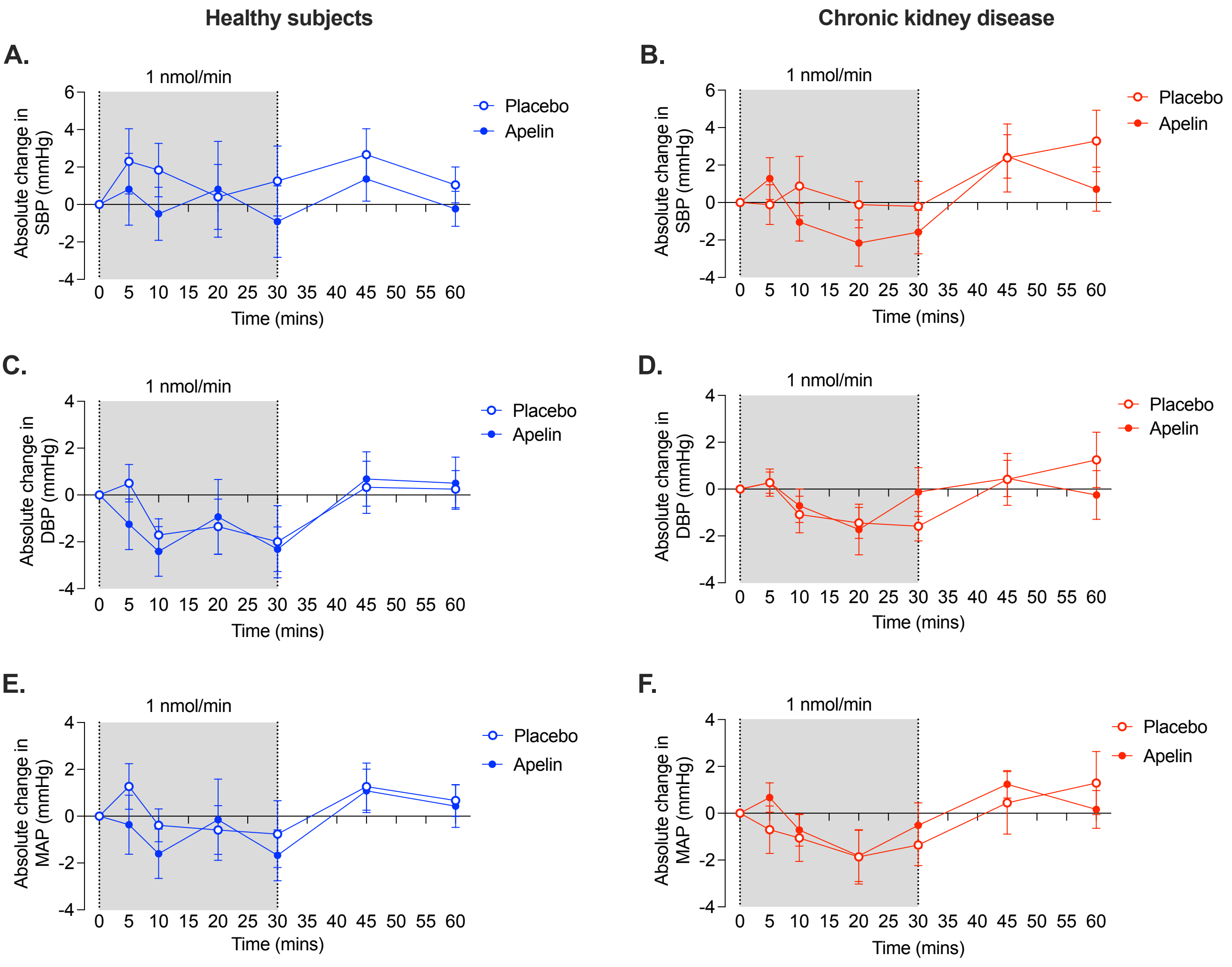

Supplementary figure 2. *Infusion of 1 nmol/min [Pyr[1]]apelin-13 did not alter blood pressure.* At a dose of 1 nmol/min [Pyr[1]]apelin-13 did not affect systolic blood pressure (SBP), diastolic blood pressure (DBP) or mean arterial pressure (MAP) in healthy subjects (A,C,E) (blue, n=12) or in patients with chronic kidney disease (B,D,F) (red, n=12). Data shown are mean +/- SEM. The grey bar represents the time during which apelin was infused. Closed circles indicate infusion of apelin; open circles indicate placebo. Analyses were by mixed effects model.

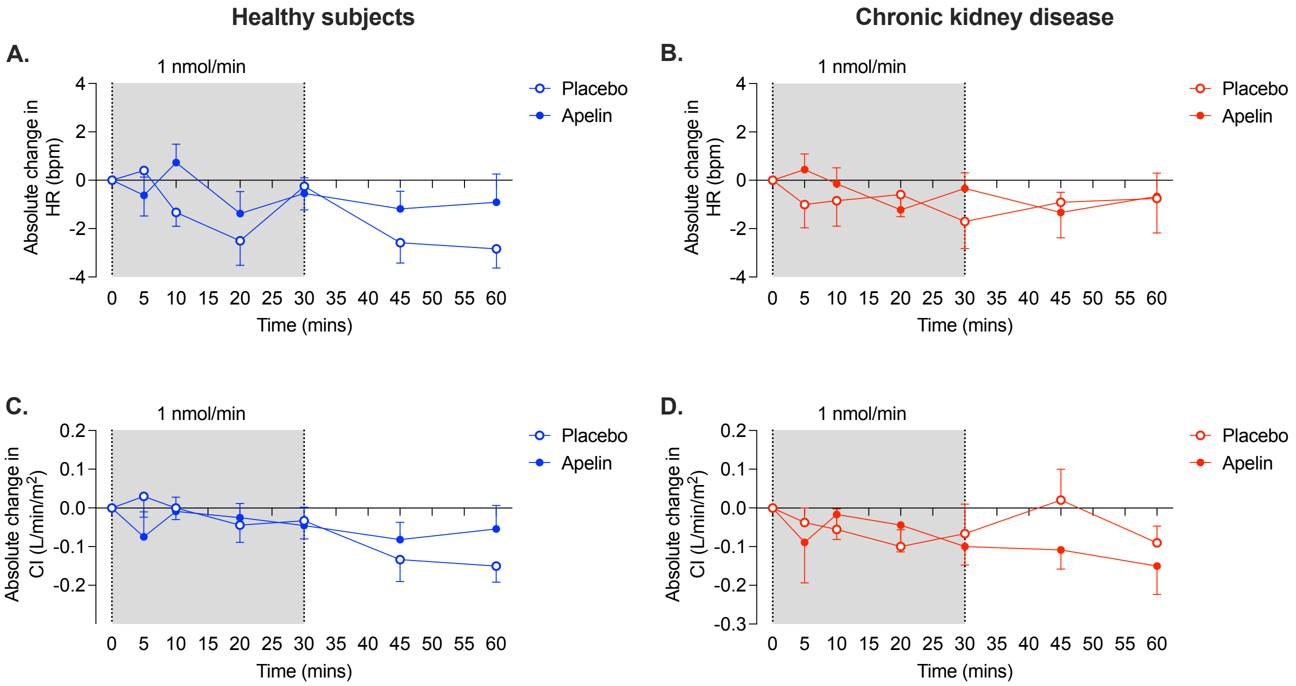

Supplementary figure 3. *Infusion of 1 nmol/min [Pyr[1]]apelin-13 did not alter heart rate or cardiac index.* In healthy subjects (blue, n=12) and patients with chronic kidney disease (CKD, red, n=12), infusion of 1 nmol/min [Pyr[1]]apelin-13 did not alter heart rate (HR; A,B) or cardiac index (CI; C, D). Data shown are mean +/- SEM. The grey bar represents the time during which apelin was infused. Closed circles indicate infusion of apelin; open circles indicate placebo. Analyses were by mixed effects model.

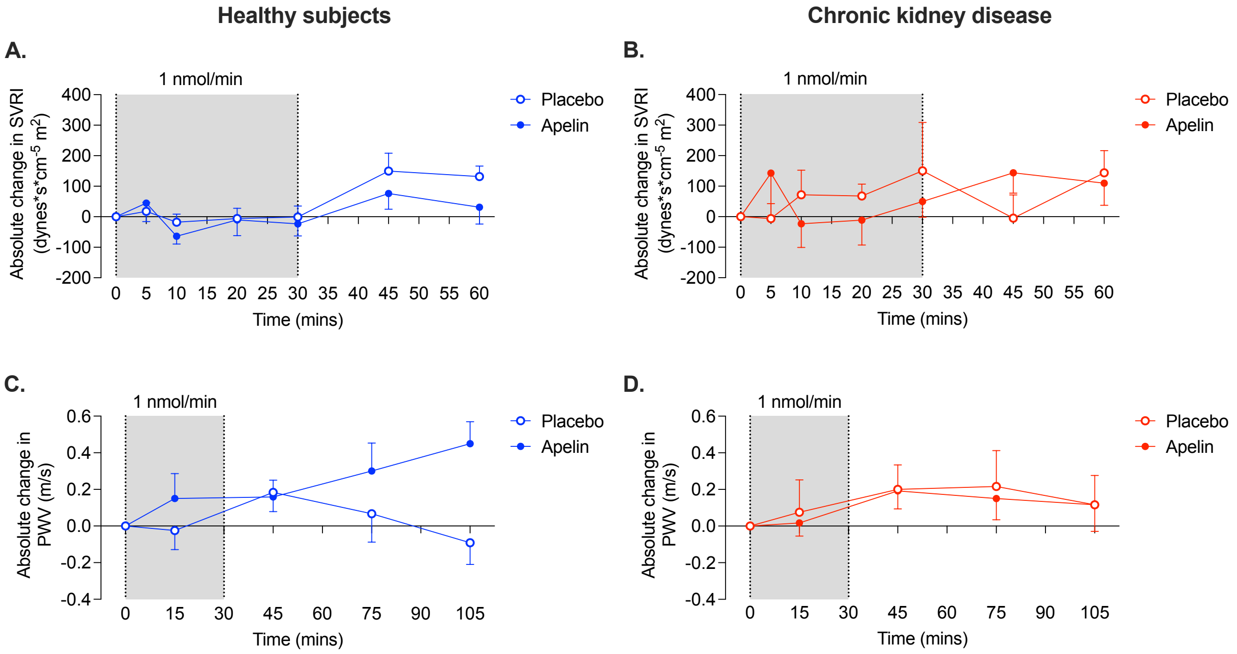

Supplementary figure 4. *Infusion of 1 nmol/min [Pyr¹]apelin-13 1 nmol/min did not alter systemic vascular resistance or arterial stiffness.* At a dose of 1 nmol/min, [Pyr¹]apelin-13 did not affect systemic vascular resistance index (SVRI; A, B) in either healthy subjects (blue, n=12) or patients with chronic kidney disease (red, n=12). Pulse wave velocity was also unaffected in health (C) and chronic kidney disease (D). Data shown are mean +/- SEM. The grey bar represents the time during which apelin was infused. Closed circles indicate infusion of apelin; open circles indicate placebo. Analyses were by mixed effects model.

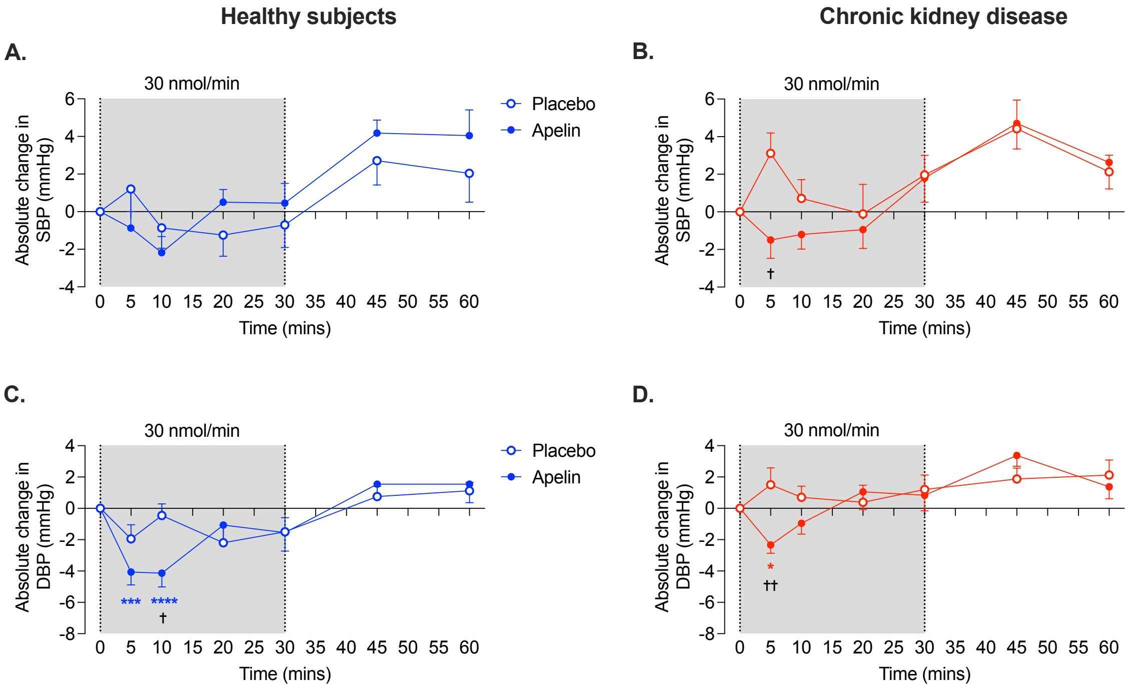

Supplementary figure 5. *[Pyr¹]apelin-13 30 nmol/min had a greater effect on diastolic blood pressure than systolic blood pressure.* Systolic blood pressure (SBP) was unaffected by 30 nmol/min [Pyr¹]apelin-13 in (A) healthy subjects (blue, n=12). (B) In patients with chronic kidney disease (red, n=12) apelin reduced SBP, where †p=0.0296 in comparison to placebo. Diastolic blood pressure (DBP) was reduced by [Pyr¹]apelin-13 in healthy subjects (C) where ***p=0.0009, ****p<0.0001 and †p=0.0204 in comparison to baseline and placebo, respectively, and in patients with CKD (D) where *p=0.046 and ††p=0.0072 in comparison to baseline and placebo, respectively. Data shown are mean +/- SEM. The grey bar represents the time during which apelin was infused. Closed circles indicate infusion of apelin; open circles indicate placebo. Analyses were by mixed effects model with Dunnett's or Šidák's multiple comparison corrections for within or between group comparisons, respectively.

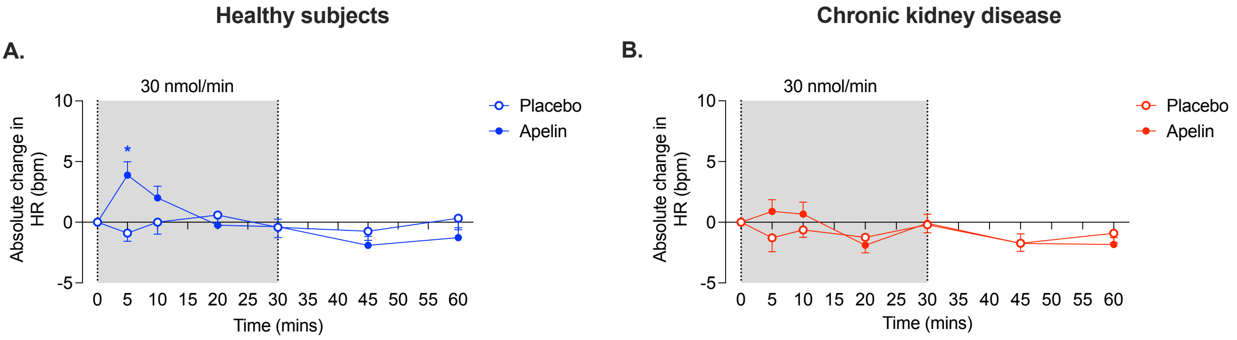

Supplementary figure 6. *Infusion of 30 nmol/min [Pyr[1]]apelin-13 increased heart rate in healthy subjects.* (A) Heart rate rose acutely in response to 30 nmol/min [Pyr[1]]apelin-13 in healthy subjects (blue, n=12) where *p=0.0295, however in patients with chronic kidney disease (red, n=12) no change was seen (B). Data shown are mean +/- SEM. The grey bar represents the time during which apelin was infused. Closed circles indicate infusion of apelin; open circles indicate placebo. Analyses were by 2-way ANOVA (CKD) or mixed effects model (health) with Dunnett's multiple comparison correction.

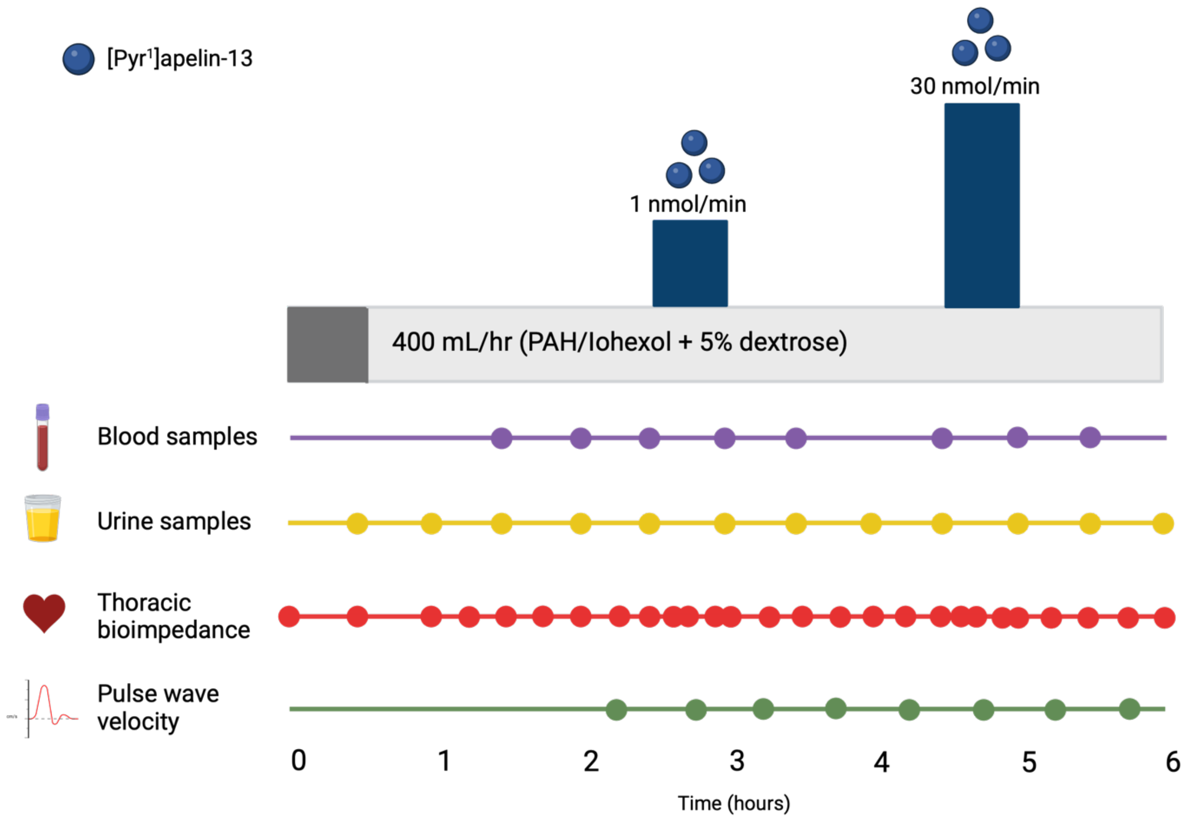

Supplementary figure 7. *Study protocol.* Participants completed a 6-hour study during which they received two 30-minute infusions of either pyroglutamated apelin-13 ([Pyr^1]apelin-13; 1 nmol/min or 30 nmol/min as indicated) or matched placebo. Following loading doses (dark grey shaded box), infusion of para-aminohippurate (PAH), iohexol and 5% dextrose (total volume infused: 400 mL/h) continued for the duration of the study. Blood and urine sampling and cardiovascular measurements were taken at regular prespecified timepoints as indicated. Supplementary figure 7 created with BioRender.com, released under a Creative Commons Attribution-NonCommercial-NoDerivs 4.0 International license https://creativecommons.org/licenses/by-nc-nd/4.0/deed.en.

STATISTICAL MODEL OUTPUTS

i. Placebo-corrected percentage change in mean arterial pressure from baseline (Fig 1A)

[Pyr[1]]apelin-13 30 nmol/min
Mixed effects model with Dunnett's multiple comparisons test

Comparison	Predicted (LS) mean difference	95% CI	SE of diff.	P value
Healthy subjects				
0 vs 5 mins	-4.0	-7.8 to -0.1	1.5	0.045
0 vs 10 mins	-2.8	-6.4 to 0.9	1.4	0.211
0 vs 20 mins	2.3	-1.6 to 6.2	1.5	0.449
0 vs 30 mins	0.7	-2.9 to 4.2	1.3	0.994
0 vs 45 mins	1.5	-2.0 to 5.1	1.3	0.746
0 vs 60 mins	1.6	-1.9 to 5.1	1.3	0.717
CKD				
0 vs 5 mins	-4.2	-8.0 to -0.3	1.5	0.027
0 vs 10 mins	-1.7	-5.2 to 1.6	1.3	0.567
0 vs 20 mins	0.6	-3.2 to 4.4	1.5	0.997
0 vs 30 mins	-0.3	-3.7 to 3.0	1.3	>0.999
0 vs 45 mins	1.2	-2.2 to 4.6	1.3	0.861
0 vs 60 mins	-0.2	-3.6 to 3.2	1.3	>0.999

ii. Placebo-corrected percentage change in systemic vascular resistance index (Fig 1B)

[Pyr[1]]apelin-13 30 nmol/min
Mixed effects model with Dunnett's multiple comparisons test

Comparison	Predicted (LS) mean difference	95% CI	SE of diff.	P value
Healthy subjects				
0 vs 5 mins	-12.0	-19.2 to -4.7	2.2	0.004
0 vs 10 mins	-8.9	-16.7 to -1.1	2.5	0.025
0 vs 20 mins	0.8	-11.6 to 13.3	3.7	>0.999
0 vs 30 mins	-2.1	-12.2 to 8.0	3.3	0.968
0 vs 45 mins	3.2	-7.2 to 13.8	3.4	0.849
0 vs 60 mins	4.0	-5.7 to 13.6	3.1	0.658
CKD				
0 vs 5 mins	-13.9	-30.7 to 2.9	4.5	0.098
0 vs 10 mins	-13.0	-23.8 to -2.2	3.5	0.018
0 vs 20 mins	-4.8	-17.9 to 8.5	3.5	0.633
0 vs 30 mins	-9.7	-20.9 to 1.6	3.6	0.100
0 vs 45 mins	-1.4	-16.2 to 13.4	4.8	>0.999
0 vs 60 mins	-1.8	-13.9 to 10.2	3.9	0.993

iii. Placebo-corrected percentage change in cardiac index (Fig 1C)

[Pyr[1]]apelin-13 30 nmol/min
Mixed effects model with Dunnett's multiple comparisons test

Comparison	Predicted (LS) mean difference	95% CI	SE of diff.	P value
Healthy subjects				
0 vs 5 mins	10.7	0.3 to 21.1	4.0	0.043
0 vs 10 mins	7.7	-1.7 to 17.2	3.6	0.154
0 vs 20 mins	3.2	-7.3 to 13.6	4.0	0.929
0 vs 30 mins	3.5	-6.0 to 12.9	3.6	0.854
0 vs 45 mins	-0.9	-10.3 to 8.6	3.6	>0.999
0 vs 60 mins	-2.0	-11.5 to 7.4	3.6	0.986
CKD				
0 vs 5 mins	9.9	-1.6 to 21.3	4.4	0.122
0 vs 10 mins	14.2	4.5 to 23.9	3.7	0.001
0 vs 20 mins	6.9	-4.6 to 18.4	4.4	0.447
0 vs 30 mins	11.5	2.1 to 20.9	3.6	0.009
0 vs 45 mins	5.5	-3.9 to 14.9	3.6	0.464
0 vs 60 mins	2.4	-7.0 to 11.8	3.6	0.971

iv. Placebo-corrected percentage change in pulse wave velocity (Fig 1D)

[Pyr[1]]apelin-13 30 nmol/min
2-way ANOVA with Dunnett's multiple comparisons test

Comparison	Mean difference	95% CI	SE of diff.	P value
Healthy subjects				
0 vs 15 mins	-6.9	-14.3 to 0.6	2.7	0.073
0 vs 45 mins	-8.4	-15.7 to -2.0	2.4	0.011
0 vs 75 mins	0.4	-7.5 to 8.4	2.9	0.998
CKD				
0 vs 15 mins	-0.2	-9.1 to 8.8	3.3	>0.999
0 vs 45 mins	-0.3	-8.1 to 7.5	2.9	0.999
0 vs 75 mins	1.9	-7.4 to 11.4	3.5	0.898

v. Placebo-corrected percentage change in effective renal blood flow from baseline (Fig 2A)

[Pyr[1]]apelin-13 1 nmol/min
Mixed effects model with Dunnett's multiple comparison test

Comparison	Predicted (LS) mean difference	95% CI	SE of diff.	P value
Healthy subjects				
0 vs 30 mins	16.3	6.0 to 26.7	4.5	0.002
0 vs 60 mins	6.2	-4.2 to 16.5	4.5	0.298
CKD				
0 vs 30 mins	9.9	0.2 to 19.6	4.2	0.045
0 vs 60 mins	10.2	1.0 to 19.6	4.1	0.034

[Pyr[1]]apelin-13 30 nmol/min
Mixed effects model with Dunnett's multiple comparison test

Comparison	Predicted (LS) mean difference	95% CI	SE of diff.	P value
Healthy subjects				
0 vs 30 mins	16.8	0.4 to 33.2	7.1	0.044
0 vs 60 mins	1.6	-14.8 to 17.9	7.1	0.964
CKD				
0 vs 30 mins	8.3	-6.7 to 23.3	6.5	0.349
0 vs 60 mins	21.2	5.8 to 36.5	6.7	0.006

vi. Placebo-corrected percentage change in glomerular filtration rate from baseline (Fig 2B)

[Pyr[1]]apelin-13 1 nmol/min
Mixed effects model with Dunnett's multiple comparison test

Comparison	Predicted (LS) mean difference	95% CI	SE of diff.	P value
Healthy subjects				
0 vs 30 mins	7.0	-1.9 to 16.0	3.9	0.141
0 vs 60 mins	4.1	-5.2 to 13.3	4.0	0.505
CKD				
0 vs 30 mins	-9.3	-17.6 to -0.9	3.6	0.028
0 vs 60 mins	-6.4	-14.6 to 1.8	3.6	0.143

[Pyr[1]]apelin-13 30 nmol/min
2-way ANOVA with Dunnett's multiple comparison test

Comparison	Mean difference	95% CI	SE of diff.	P value
Healthy subjects				
0 vs 30 mins	1.5	-16.0 to 19.1	6.7	0.964
0 vs 60 mins	-7.5	-23.9 to 8.1	6.0	0389
CKD				
0 vs 30 mins	-14.4	-22.0 to -6.7	3.0	0.009
0 vs 60 mins	-5.1	-13.0 to 2.9	3.1	0.224

vii. Placebo-corrected absolute change in effective filtration fraction from baseline (Fig 2C)

[Pyr[1]]apelin-13 1 nmol/min
Mixed effects model with Dunnett's multiple comparison test

Comparison	Predicted (LS) mean difference	95% CI	SE of diff.	P value
Healthy subjects				
0 vs 30 mins	-1.5	-3.2 to 0.2	0.7	0.097
0 vs 60 mins	-0.1	-1.8 to 1.7	0.8	0.992
CKD				
0 vs 30 mins	-3.0	-4.6 to -1.4	0.7	0.0002
0 vs 60 mins	-3.3	-4.8 to -1.7	0.7	<0.0001

[Pyr[1]]apelin-13 30 nmol/min
Mixed effects model with Dunnett's multiple comparison test

Comparison	Predicted (LS) mean difference	95% CI	SE of diff.	P value
Healthy subjects				
0 vs 30 mins	-2.4	-4.6 to -0.1	2.3	0.045
0 vs 60 mins	-1.5	-3.8 to 0.9	1.5	0.264
CKD				
0 vs 30 mins	-4.2	-6.3 to -2.1	4.6	<0.0001
0 vs 60 mins	-5.2	-7.4 to -3.0	5.5	<0.0001

viii. Absolute change in protein excretion from baseline in CKD (Fig 2D)

[Pyr[1]]apelin-13 1 nmol/min
Mixed effects model with Šidák's multiple comparison test

Comparison	Predicted (LS) mean difference	95% CI	SE of diff.	P value
Placebo				
0 vs 30 mins	18.6	-49.9 to 87.0	27.0	0.723
0 vs 60 mins	-17.8	-39.5 to 3.8	8.5	0.107
Apelin				
0 vs 30 mins	-57.3	-98.4 to -16.2	16.0	0.009
0 vs 60 mins	-47.4	-77.4 to -17.4	11.7	0.004

[Pyr[1]]apelin-13 30 nmol/min
Mixed effects model with Šidák's multiple comparison test

Comparison	Predicted (LS) mean difference	95% CI	SE of diff.	P value
Placebo				
0 vs 30 mins	6.3	-29.9 to 42.3	15.6	0.903
0 vs 60 mins	-6.7	-42.9 to 29.5	15.6	0.891
Apelin				
0 vs 30 mins	-51.2	-87.4 to -15.1	15.6	0.004
0 vs 60 mins	-34.0	-71.6 to 3.12	16.0	0.078

ix. Placebo-corrected percentage change in urine sodium excretion (Fig 3A)

[Pyr[1]]apelin-13 1 nmol/min
Mixed effects model with Dunnett's multiple comparison test

Comparison	Predicted (LS) mean difference	95% CI	SE of diff.	P value
Healthy subjects				
0 vs 30 mins	32.2	13.9 to 50.1	8.0	0.001
0 vs 60 mins	7.6	-12.0 to 27.2	8.5	0.583
CKD				
0 vs 30 mins	18.5	1.4 to 35.7	7.5	0.033
0 vs 60 mins	26.9	10.2 to 43.6	7.3	0.001

[Pyr[1]]apelin-13 30 nmol/min
Mixed effects model with Dunnett's multiple comparison test

Comparison	Predicted (LS) mean difference	95% CI	SE of diff.	P value
Healthy subjects				
0 vs 30 mins	14.4	-4.2 to 33.0	7.1	0.127
0 vs 60 mins	11.9	-7.1 to 30.9	7.3	0.230
CKD				
0 vs 30 mins	10.9	6.9 to 28.6	7.0	0.249
0 vs 60 mins	28.6	9.5 to 47.6	7.4	0.006

x. Placebo-corrected percentage change in free water clearance (Fig 3B)

[Pyr[1]]apelin-13 1 nmol/min
Mixed effects model with Dunnett's multiple comparison test

Comparison	Mean difference	95% CI	SE of diff.	P value
Healthy subjects				
0 vs 30 mins	31.4	2.2 to 60.6	10.9	0.037
0 vs 60 mins	12.4	-12.4 to 37.2	9.0	0.344
CKD				
0 vs 30 mins	9.9	1.1 to 18.6	3.4	0.029
0 vs 60 mins	14.2	2.5 to 25.8	4.6	0.019

[Pyr[1]]apelin-13 30 nmol/min
Mixed effects model with Dunnett's multiple comparison test

Comparison	Mean difference	95% CI	SE of diff.	P value
Healthy subjects				
0 vs 30 mins	10.5	-16.6 to 37.5	10.1	0.547
0 vs 60 mins	12.4	-17.6 to 42.5	10.9	0.493
CKD				
0 vs 30 mins	4.7	-13.1 to 22.6	6.8	0.753
0 vs 60 mins	19.9	2.5 to 37.4	6.4	0.028
