# Supplementary material for: Cardiovascular and renal effects of apelin in chronic kidney disease: a randomised, double-blind, placebo-controlled, crossover study
Source: Nat Commun. 2024 Oct 14;15:8387. doi: 10.1038/s41467-024-52447-7 (PMC11473822; doi:10.1038/s41467-024-52447-7)
Supplement: Supplementary file 3 — Reporting Summary [file 41467_2024_52447_MOESM3_ESM.pdf]

nature portfolio

Corresponding author(s): Neeraj Dhaun

Last updated by author(s): 1 September 2024

Reporting Summary

Nature Portfolio wishes to improve the reproducibility of the work that we publish. This form provides structure for consistency and transparency in reporting. For further information on Nature Portfolio policies, see our Editorial Policies and the Editorial Policy Checklist.

Statistics

For all statistical analyses, confirm that the following items are present in the figure legend, table legend, main text, or Methods section.

n/a	Confirmed	
[ ]	[x]	The exact sample size (*n*) for each experimental group/condition, given as a discrete number and unit of measurement
[ ]	[x]	A statement on whether measurements were taken from distinct samples or whether the same sample was measured repeatedly
[ ]	[x]	The statistical test(s) used AND whether they are one- or two-sided<br>*Only common tests should be described solely by name; describe more complex techniques in the Methods section.*
[ ]	[x]	A description of all covariates tested
[ ]	[x]	A description of any assumptions or corrections, such as tests of normality and adjustment for multiple comparisons
[ ]	[x]	A full description of the statistical parameters including central tendency (e.g. means) or other basic estimates (e.g. regression coefficient) AND variation (e.g. standard deviation) or associated estimates of uncertainty (e.g. confidence intervals)
[ ]	[x]	For null hypothesis testing, the test statistic (e.g. *F*, *t*, *r*) with confidence intervals, effect sizes, degrees of freedom and *P* value noted<br>*Give P values as exact values whenever suitable.*
[x]	[ ]	For Bayesian analysis, information on the choice of priors and Markov chain Monte Carlo settings
[x]	[ ]	For hierarchical and complex designs, identification of the appropriate level for tests and full reporting of outcomes
[ ]	[x]	Estimates of effect sizes (e.g. Cohen's *d*, Pearson's *r*), indicating how they were calculated

Our web collection on statistics for biologists contains articles on many of the points above.

Software and code

Policy information about availability of computer code

Data collection	No data collection software was used
Data analysis	Data analysis was performed on Prism, version 9.4.1, GraphPad Software Inc, San Diego, USA

For manuscripts utilizing custom algorithms or software that are central to the research but not yet described in published literature, software must be made available to editors and reviewers. We strongly encourage code deposition in a community repository (e.g. GitHub). See the Nature Portfolio guidelines for submitting code & software for further information.

Data

Policy information about availability of data

All manuscripts must include a data availability statement. This statement should provide the following information, where applicable:

- Accession codes, unique identifiers, or web links for publicly available datasets
- A description of any restrictions on data availability
- For clinical datasets or third party data, please ensure that the statement adheres to our policy

Anonymized individual participant source data are provided with this manuscript.

Research involving human participants, their data, or biological material

Policy information about studies with human participants or human data. See also policy information about sex, gender (identity/presentation), and sexual orientation and race, ethnicity and racism.

Reporting on sex and gender	Sex was determined by self-reporting. Due to the sample size, no sex-based analysis was performed.
Reporting on race, ethnicity, or other socially relevant groupings	Due to the nature of this study, race and ethnicity have not been reported.
Population characteristics	Human adults (>18 years) who were healthy or who had chronic kidney disease.
Recruitment	An established Renal Research team recruited patients with chronic kidney disease from within NHS Lothian. Patients were approached if they fulfilled the inclusion criteria with verbal and written information provided. Informed consent was taken at a second visit >7 days later. This may have selected patients who were more engaged with healthcare services and they may not be fully representative of typical patients with chronic kidney disease. Age- and sex-matched healthy subjects were identified from local advertisement and locally approved volunteer databases. Verbal and written information was again provided with informed consent taken at a subsequent visit. Given the engagement with research this may have selected a particularly healthy control population.
Ethics oversight	All studies were carried out at the University of Edinburgh according to the principles of the Declaration of Helsinki. They were approved by the South East Scotland Research Ethics committee (ref. 18/SS/0145) and ACCORD and were performed with written informed consent from each subject.

Note that full information on the approval of the study protocol must also be provided in the manuscript.

Field-specific reporting

Please select the one below that is the best fit for your research. If you are not sure, read the appropriate sections before making your selection.

[x] Life sciences [ ] Behavioural & social sciences [ ] Ecological, evolutionary & environmental sciences

For a reference copy of the document with all sections, see nature.com/documents/nr-reporting-summary-flat.pdf

Life sciences study design

All studies must disclose on these points even when the disclosure is negative.

Sample size	As apelin has not been given to patients with chronic kidney disease before, the sample size in this study was determined using data from previous studies of apelin in healthy subjects and patients with heart failure. The coprimary endpoints used were systemic vascular resistance and effective renal blood flow. A sample size of 12 participants per group (12 chronic kidney disease, 12 healthy subjects) would have 85% power to detect a 10% reduction in systemic vascular resistance index and a 15% increase in effective renal blood flow in response to apelin in comparison to placebo, with a 2-sided significance level of 5%.
Data exclusions	Prespecified criteria were in place for data exclusion. With respect to iohexol and para-aminohippurate clearance, individual data points were excluded if baseline urinary flow rate was not stable as this is essential for data accuracy. With respect to cardiovascular data, there was a requirement for each variable to be reproducible on repeated measures with specific criteria for each (e.g. blood pressure 2 sequential readings within 10mmHg). If measurements did not meet this criteria they were repeated. If repeat measurements remained unstable then readings from this timepoint were occasionally omitted at the discretion of the investigator in the overall interests of the participant's study (to avoid stressors).
Replication	Cardiovascular measurements were repeated as described within the methods at each timepoint on each study visit. Blood pressure was recorded as an average of two measures from the right arm with <10mmHg difference. Pulse wave velocity was recorded as an average of two readings with <0.5m/s variability. Impedance cardiography measurements were taken as an average of two readings with the difference in systemic vascular resistance index required to be <10%. As above, if these criteria were not achieved at a set timepoint then measurements were repeated. If stable measurements were not achieved then data from that timepoint could be omitted at the discretion of the investigator in the overall interest of the study.
Randomization	A randomization schedule for participant study visits (apelin OR placebo) was generated prior to study recruitment for each group (healthy subjects and patients with chronic kidney disease). Once recruited, the study nurse randomly allocated participants; the investigator remained blinded.
Blinding	This was a double-blinded study. Following recruitment all study investigators were blinded to treatment allocation at each study visit (apelin or placebo) and remained so until all data collection and laboratory measurements were complete. Data were unblinded once the analysis was complete.

Reporting for specific materials, systems and methods

We require information from authors about some types of materials, experimental systems and methods used in many studies. Here, indicate whether each material, system or method listed is relevant to your study. If you are not sure if a list item applies to your research, read the appropriate section before selecting a response.

Materials & experimental systems

n/a	Involved in the study
☒	☐ Antibodies
☒	☐ Eukaryotic cell lines
☒	☐ Palaeontology and archaeology
☒	☐ Animals and other organisms
☐	☒ Clinical data
☒	☐ Dual use research of concern
☒	☐ Plants

Methods

n/a	Involved in the study
☒	☐ ChIP-seq
☒	☐ Flow cytometry
☒	☐ MRI-based neuroimaging

Clinical data

Policy information about clinical studies

All manuscripts should comply with the ICMJEguidelines for publication of clinical research and a completedCONSORT checklist must be included with all submissions.

Clinical trial registration	NCT03956576
Study protocol	The study protocol has been provided with the submitted manuscript. A copy of the study protocol will be provided upon reasonable request.
Data collection	Collected at the University of Edinburgh. Study recruitment between April 2021 - November 2022. All study visits were carried out in the University of Edinburgh Clinical Research Centre, Western General Hospital, Edinburgh, between May 2021 - December 2022.
Outcomes	All endpoints were prespecified at the time of the study design. Apelin has not been given to patients with chronic kidney disease so these studies were based on data from healthy subjects and patients with heart failure. The co-primary endpoints were change in systemic vascular resistance index and effective renal blood flow. A sample size of 12 participants per group (12 chronic kidney disease, 12 healthy subjects) would have 85% power to detect a 10% reduction in systemic vascular resistance index and a 15% increase in effective renal blood flow in response to apelin in comparison to placebo, with a 2-sided significance level of 5%. Secondary outcomes were changes in cardiac output, blood pressure, pulse wave velocity, glomerular filtration rate, natriuresis, free water clearance and urinary protein excretion.

Plants

Seed stocks	*Report on the source of all seed stocks or other plant material used. If applicable, state the seed stock centre and catalogue number. If plant specimens were collected from the field, describe the collection location, date and sampling procedures.*
Novel plant genotypes	*Describe the methods by which all novel plant genotypes were produced. This includes those generated by transgenic approaches, gene editing, chemical/radiation-based mutagenesis and hybridization. For transgenic lines, describe the transformation method, the number of independent lines analyzed and the generation upon which experiments were performed. For gene-edited lines, describe the editor used, the endogenous sequence targeted for editing, the targeting guide RNA sequence (if applicable) and how the editor was applied.*
Authentication	*Describe any authentication procedures for each seed stock used or novel genotype generated. Describe any experiments used to assess the effect of a mutation and, where applicable, how potential secondary effects (e.g. second site T-DNA insertions, mosiacism, off-target gene editing) were examined.*
